# Nursing students’ development of using physical assessment in clinical rotation—a stimulated recall study

**DOI:** 10.1186/s12912-022-00879-1

**Published:** 2022-05-10

**Authors:** Kirsten Røland Byermoen, Tom Eide, H. Ösp Egilsdottir, Hilde Eide, Lena Günterberg Heyn, Anne Moen, Espen Andreas Brembo

**Affiliations:** 1 Centre for Health and Technology, Faculty of Health and Social Sciences, University of South-Eastern Norway, Grønland 58, 3045 Drammen, Norway; 2 Department of Nursing Science, Faculty of Medicine, University of Oslo, Forskningsveien 2B, 0371 Oslo, Norway

**Keywords:** Physical examination, Nursing assessment, Clinical reasoning, Clinical competence, Education nursing, Student placement, Stimulated recall interview

## Abstract

**Background:**

The overall aim of this study was to explore third-year bachelor nursing students’ stimulated recall reflections on their physical assessment competence development. The choice of learning strategies in nursing education seems to have great impact on nursing students’ use of physical assessment skills while in clinical rotation. There is a need to explore nursing students’ learning processes related to the use of physical assessments.

**Methods:**

Explorative qualitative design using a triangulation of data collection methods. Nine final-year nursing students’ physical assessment performances during patient encounters were audio-taped and observed. Shortly after, an individual stimulated recall interview based on the audio-recorded patient encounter and observation notes was conducted. A two-fold analysis was conducted: 1) analysis of students’ performed assessments, and 2) phenomenological hermeneutical analysis of the stimulated recall interviews.

**Results:**

Nursing students assessments shifted from a checklist approach to a symptom-based, more holistic and person-centred approach, emphasizing conversation as part of their assessments. The nursing students also reported that a safe and stimulating learning environment was a prominent feature for their continuing development. Learning from skilled role models with expectations to them using physical assessment skills facilitated their continuing skills appliance, interprofessional communication and reflective practice.

**Conclusions:**

This study contribute with a novel, comprehensive and in-depth description of what influenced nursing students’ learning processes experiences of using physical assessment skills during clinical rotation. The results reveal the need for targeted course designs by implementing scaffolded learning activities in practical and theoretical courses aimed at strengthening students’ learning of physical assessment skills—building upon and emphasizing their prior knowledge and competence, which may lead to more confident registered nurses and promote patient safety in different health care contexts. We propose using stimulated recall systematically as a novel reflective learning activity in nursing education to foster clinical reasoning and metacognition skills and achieve deep learning.

## Background

Health reforms aim to strengthen primary health care and reduce hospitalization [1, 2]. With an ageing global population, comorbidity and complex health problems, modern health reforms address increasingly complex challenges in primary health care. This increased complexity makes it more demanding for nurses to perform thorough, valid assessments of patients’ current health status, as well as to anticipate future clinical changes. Clinical nursing skills and competence are therefore in great demand. The implementation of early warning score (EWS) systems and advances in nurses’ competence within physical assessment skills represent practical and proactive approaches that help nurses detect clinical deterioration and prevent complications [3, 4]. Newly graduated nurses are expected to have sufficient clinical competence to secure patient safety and meet quality standards of care [5]. Hence, there is a need to readdress undergraduate nursing curricula regarding clinical competence, to prepare nursing students for demanding challenges and increased nursing competence needs [6].

Physical assessment are core clinical competences in nursing practice, forming the basis of nursing students’ preparedness for potentially complex patient encounters as registered nurses [4]. Obtaining subjective and objective data through physical assessment enables nurses to provide more accurate assessments of patients’ medical and clinical conditions [6]. A standardized interdisciplinary assessment scheme provides a standardized terminology across health care disciplines that can increase the overall quality of care for patients in complex clinical encounters [7].

Studies has over the last decade reported that nursing students and newly graduated nurses do not perform all of their learned physical assessment skills during clinical encounters [8–11]. Several factors limit their use of physical assessment skills, such as role ambiguity, reliance on technology, collegial support and culture, practice variations across specialities, lack of confidence and knowledge, and over-teaching using the biomedical model [12]. Studies indicate that implementation of physical assessment courses in nursing education increases students’ confidence, assertiveness and self-esteem [13–16].

In an earlier study, we examined the implementation of a physical assessment skills curriculum at our university, and proposed a model to scaffolding of physical assessment practice over the three-year education [8]. Using self-reports, we also identified which skills were frequently, or less frequently, used in clinical rotation. Assessments of the heart and peripheral circulatory system (emphasizing inspection, palpation, blood pressure and blood oxygen) were most used, and assessments of neurology, percussion and auscultation in abdominal, respiratory and circulatory systems were least used. Factors that hindered practice were lack of support in the learning environment and preceptors not using physical assessment skills. We also performed an observation study combined with a stimulated recall interview (SRI) to gain more in-depth knowledge about how third-year nursing students learn and practise specific physical assessment skills—and to explore facilitators and barriers in the learning process [17]. Facilitating factors included peer learning; students’ ability to transform experiences from prior clinical encounters; their ability to articulate reasoning in relation to human bioscience; and engaged role models who expected students to perform physical assessment. A lack of role models and few opportunities to practise were reported as barriers to assessment skills practice, as was students’ doubt about its impact.

To our knowledge, there are no longitudinal follow-up studies that explore students’ development of physical assessment over time. Moreover, nursing students’ learning process around physical assessment remains under-researched, specifically with regards to promoting and supporting their development of competence in this area.

## Methods

### Aim and research questions

This paper is part of a longitudinal study exploring students’ competence development of physical assessment during several clinical rotations in undergraduate nursing education. The overall aim of this paper is to explore third-year bachelor nursing students’ 1) physical assessment practice, and 2) stimulated recall reflections on their competence development. The following research questions guided the analysis and interpretation of the results:

- Which physical assessment skills do students apply during patient encounters and why?
- What do the students experience as important learning environment factors influencing their learning process?
- Which learning strategies do the students apply during clinical rotation to integrate physical assessment as a routine?

### Design

**Fig. 1:**
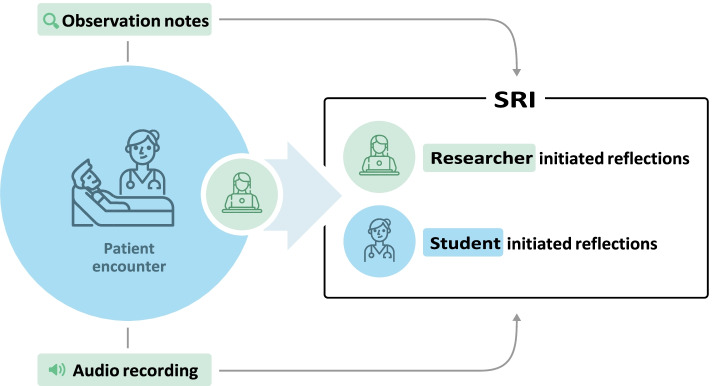
Visual model of data collection

This is a follow-up study, utilizing the same data collection method and student sample from a previous study [17]. The study has an explorative qualitative design and uses a triangulation of data collection methods, as illustrated in Fig. 1: 1) observation including notes and audio-recordings of nursing students during a patient encounter, and 2) SRIs based on the audio-recorded patient encounter. Exploratory design explores the full nature of a phenomena, to shed light on various factors and underlying processes [18]. The chosen study design was selected to capture perspectives of nursing students’ perceived experiences and enable in-depth observation of which physical assessment skills they used.

### Data collection and sample

Data were collected in March 2019, during week 7 or 8 of the programme’s final clinical rotation (a total of 8 weeks/240 hours). Fifteen final-semester nursing students who had their final clinical rotation in a predetermined nursing home and home care location were invited to contact the clinical rotation coordinator if they were interested in taking part in the study. The first author then provided them with detailed oral and written information. Nine students agreed to participate. A total of eight patient encounter observations and nine interviews were conducted.

Participating nursing students ranged from 23 to 50 years of age, with an average of 31. One participant was male; three did not have Norwegian as their native language; five had prior health-related work experience; and six were working as auxiliary nurses during their education (Table 1).

**Table 1:** Characteristics of the student sample

Background information	Age-range^1^	Health-related work experience prior to education start (years)	Health-related work experience during education (shifts/week)	Number of PAS performed during patient encounter (*N* = 44)	Patient encounter time (minutes)	SRI time (minutes)
Student 1	1	2.5	2	8	24	42
Student 2	2	0	0	14	8	43
Student 3	1	0	2.5	12	13	27
Student 4	3	0.5	0	24	16	44
Student 5	1	6	2.5	–	–	17
Student 6	1	0	2	24	17	47
Student 7	1	6	2.5	14	27	34
Student 8	3	0	0	5	17	30
Student 9	2	5	2	13	16	31

^1^Age range: 1: 23–30 years old; 2: 31–40 years old; 3: 41–50 years old

^*2*^*PAS* Physical assessment skills

Eight patients also agreed to participate and signed the consent form before the researcher was introduced. One student and preceptor were unable to recruit a patient: nevertheless, this student had previously performed physical assessment of a patient prior to the researcher’s arrival, and wanted to share experiences from this assessment and their own development in the use of physical assessment in an interview.

Participating patients ranged in age from 60 to 89, with an average age of 79. Two were male; all had Norwegian as their native language; one lived permanently in a nursing home; three received in-house rehabilitation; and four received home care.

### Observation of students’ physical assessment performance

Empirical data from the patient encounter comprise the following: 1) direct observation by the first author with observational notes and audio-recording of the patient encounter, and 2) audio-recordings of student–patient interaction during the encounter. Structured observational notes based on the physical assessment curriculum (Table 2) were used to systematically and objectively assess which physical assessment skills were applied.

**Table 2:** Overview of physical assessment skills curriculum

Organ system	Physical assessment skills curricula
**Heart and peripheral circulatory system**	Inspect extremities for skin colour/hair growth
	Palpate distal pulses
	Count pulses
	Palpate for oedema
	Palpate and inspect capillary refill
	Estimate turgor
	Evaluate extremities for skin sensation
	Assess fine motor skills
	Take blood pressure
	Auscultate heart sounds
	Auscultate carotid artery
**Respiratory system**	Inspect thorax for shape, breathing effort
	Inspect thorax for skin colour/scar
	Palpate thorax wall for thoracic expansion and vocal fremitus
	Percuss lungs
	Auscultate lungs
	Assess SpO_2_^1^
**Abdominal system**	Inspect abdomen
	Auscultate abdomen for bowel sounds
	Abdominal palpation
	Percuss the abdomen
	Percuss for kidney tenderness
**Neurological system**	Evaluate mental status
	Evaluate CN I–XII^2^
	Evaluate muscle strength, atrophy, tone
	Evaluate sensation of touch
	Assess coordination and balance
	Evaluate patella and plantar reflexes

^*1*^*SpO*_*2*_*:* blood oxygen level*,*^*2*^*CNI-XII Cranial nerves numbers 1–12*

The students were encouraged to perform assessments they considered relevant during that encounter. All patient encounters differed, as the invited patients had different diagnoses and health situations and were in either home care or in nursing homes. Direct observation by the researcher was essential to complement the audio-recordings, in order to observe 1) non-verbal communication, and 2) performance of the assessments.

### Individual stimulated recall interview (SRI)

The first author conducted an individual SRI with each student shortly after the patient encounter to ensure immediate recall of the clinical situation and contextual elements. SRIs provide an in-depth exploration of the patient encounter through the students’ reflections [19]. The SRI took place in a private room at the clinical rotation site. At the nursing homes, the SRIs were conducted within 5 minutes after the patient encounter; SRIs in the home care setting were conducted once the students had returned to their rotation base (within approximately 20 minutes).

The SRI has been found to be a reliable methodology [20] that is well-suited to explorations of nursing students’ behaviours and reflections on their own actions while performing physical assessment. In the present study, the SRI entailed interviewing students while listening to audio-recordings of the patient encounter. Students were encouraged to pause the audio-recording whenever they felt like sharing their reflections. When events occurred that called for further exploration and reflection, the researcher would suggest that they pause the recording if the student had not already done so.

A thematic interview guide included questions that explored 1) perceptions about performing physical assessment during the patient encounter, 2) factors influencing the use of physical assessment, 3) students’ reasons for performing the specific assessment skills in that particular situation, and 4) students’ own experience of their development during their final clinical rotation. The observational notes from each patient encounter enabled the researcher to point out areas to discuss with the student, of their performance (or not performance) of specific assessment skills.

### Data analysis

The data analysis involved 1) analysis of the students’ performance of physical assessment during the patient encounter, and 2) analysis of the SRIs.

### Analysis of nursing students’ performed physical assessment

The analysis of students’ performed assessment was based on the researcher’s observations and the student’s reasoning during the SRI regarding their performed assessment. The data from the patient encounter consist of a report of the patient’s clinical condition and medical diagnosis and the structured observation notes about the student’s performed physical assessment. The evaluation consists of the nursing student’s use of physical assessment skills during the encounter: i.e., which assessment skills were performed, how skills were applied and the student’s reasoning during the SRI.

### Analysis of the stimulated recall interviews

**Fig. 2:**
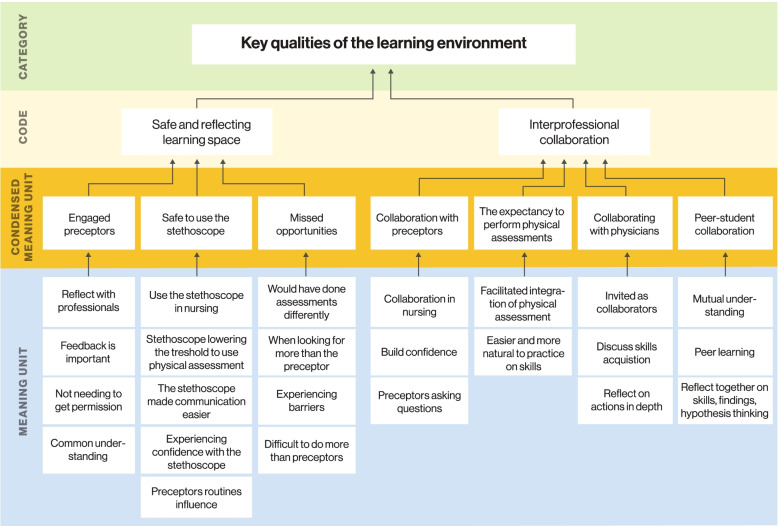
Analysis from meaning units to categories

Audio-recordings from each SRI were transcribed verbatim. Although both patients’ and students’ voices from the encounter were present in the audio-recordings played during the SRI, it was only the interaction between the student and the researcher that was transcribed. The qualitative analysis of the text followed Lindseth and Norberg’s phenomenological hermeneutical approach, moving repeatedly through the three stages of naïve reading, structural analysis and comprehensive understanding, as described below [21]. We were also inspired by Brinkman and Kvale’s description of the systematic process of moving from meaning units and condensed meaning units, to codes and categories [22] as shown with an example in Fig. 2.

KRB, TE and EAB were responsible for the analysis. We first read all interviews in a reflexive, naïve and inductive manner, to gain impressions of the meaning of the text as a whole. At this stage, we sought an overall understanding of the students’ learning process and how they experienced their own performance of physical assessment. KRB then structured the text into meaning units by posing analytical or productive questions to the text. The next steps of analysis were highly iterative and hermeneutic in nature and involved a cycle of repeated reading, processes of decontextualization and recontextualizations of empirical excerpts, and frequent discussions between the three researchers [21]. Meaning units, codes and categories were assessed several times and validated or refuted based on a joint understanding of the empirical excerpts. Codes were derived from the meaning units once they were considered to reflect and represent meaning across the data. In the final step, the main categories were developed [22].

### Research ethics

The Norwegian Centre for Research Data (NSD) approved the study (Project No. 196758). The involved municipalities and institutional leaders granted their approval before patient recruitment began. The students and preceptors approached eligible patients with written and oral information about the aim of the study (i.e., direct observation and audio-recording). Only patients who were able to consent to participation were invited. The researcher was first introduced to the patient after a signed consent form was obtained. No data were collected from the patients, other than the diagnosis and medical condition presented by the student.

The students knew the researcher primarily as a member of the faculty in the Bachelor of Nursing programme at the university. Throughout the study, the students were given oral and written information reminding them that their participation and performance in the study would not be evaluated as a part of the clinical rotation. The faculty member responsible for the formal evaluation of the student did not discuss the students’ performance or reflections with the researchers.

As the imbalanced power relationship between researcher and student requires attention [23, 24], the researcher was conscious of the students’ experiences when they were asked to articulate and reflect upon their own skills application, competence and knowledge. The researcher worked to build rapport, use careful wording, and attend to their own and the students’ body language to reduce discomfort [25, 26]. To decrease the risk of observer bias, all students were explicitly encouraged to correct the researcher during the SRI, while listening to the audio-recordings and reflecting on their performance.

## Results

In this section, we first present descriptions of students’ performed assessment skills and reasoning during the patient encounters that were based on the observations. We then describe findings from the SRIs.

### Nursing students’ performed assessments and reasonings

A common strategy for all students was to converse with the patients to perform a quick assessment of their current clinical condition. The conversations revealed possible clinical changes in the patients’ health condition, which helped most students determine which physical assessment skills to perform. Consequently, most students did not perform all of the skills they had learned, instead focusing on the patients’ concerns and symptoms through a symptom-based approach. Half of the students used a head-to-toe approach that supported a systematic flow of the performed assessment skills.

Our analysis of the observations showed different competency levels for students’ clinical reasoning regarding their performed physical assessment. Students who referenced prior clinical experiences during the current patient encounter to determine which skills to use also performed more targeted physical assessment (Table 3). Students who did not do so were less able to articulate why they performed specific assessments, nor discover relevant skills to perform during the encounter.

**Table 3:** Evaluation of nursing students’ performed assessment skills

***Patient clinical condition***	***Patient medical diagnosis***	***Nursing students’ performed physical assessment skills***	***Evaluation of student performance***
Female 74 years old. Receives home nursing follow-up after hip surgery dexter, 2 months ago	Recurrent hip luxations—surgical treatment several times, COPD^1^, hypertension	**Heart and peripheral circulation:** Inspect extremities for skin colour/hair growth, palpate distal pulses, palpate for oedema, take blood pressure, auscultate heart sounds **Thorax:** Inspect thoracic wall for shape and breathing effort, inspect thorax for skin colour/scar, auscultate lungs, assess SpO2^2^ **Abdomen:** Take history on bowel function and perform inspection inside mouth on mucosa and teeth **Neurology:** Take history on neurological status/movement in legs and feet, assess mental status, cranial nerves II	Student initiates assessment through conversation. Performs suitable skills related to patient’s clinical and medical diagnosis. Takes a head-to-toe approach, where patient’s symptoms are in focus for skills application. Student references prior clinical encounters to support clinical reasoning for performed assessment skills. The current patient encounter is a starting point for reflections, and reflections go beyond the actual assessment on the audio-recording.
Female 60 years old. Receives home nursing for maintenance and care of suprapubic catheter. Severe spinal pain—pain management. Elbow wound treatment	Several spinal surgeries—with complications, unable to hold torso upright	**Heart and peripheral circulation:** Inspect thorax for shape, breathing effort, assess pain sensation **Abdomen:** Inspect abdomen/skin around the suprapubic catheter, light abdominal palpation **Neurology:** Assess mental status	Student initiates encounter through conversation. No assessment skills were explicitly performed. Left out relevant assessments related to heart and peripheral circulation, pain management and abdominal assessment due to complications in torso. Student articulates which assessments and the reasoning for why they were performed.
Female 89 years old. Receives home nursing due to age and assistance during morning care	Rheumatoid arthritis, Sjögren syndrome, heart failure, hypertension, pneumonia 12 weeks ago, ear infection 5 weeks ago	**Heart and peripheral circulation:** Inspect extremities for skin colour/hair growth, palpate distal pulses, palpate for oedema, estimate skin fold, assess pain sensation **Thorax:** Inspect thorax for shape and breathing effort, inspect thorax for skin colour/scar, palpate thorax wall for thoracic expansion and vocal fremitus, percuss lungs, auscultate lungs, assess SpO_2._^2^ **Abdomen:** Perform inspection inside mouth on mucosa and teeth **Neurology:** Assess mental status	Student initiates assessment through conversation. Performs suitable skills related to patient’s clinical and medical diagnosis. Takes a symptom-based approach through history-taking and conversation. Student references prior clinical encounters to support clinical reasoning for performed assessment skills. The current patient encounter is a starting point for reflections, and reflections go beyond the actual assessment on the audio-recording.
Female 94 years old. Admitted to nursing home due to failure to thrive	Asthma, former PCI^3^ intervention, former breast cancer and uterus cancer—no complications after surgery	**Heart and peripheral circulation:** Inspect extremities for skin colour/hair growth, palpate distal pulses, palpate for oedema, palpate and inspect capillary refill, evaluate extremities for skin sensation, assess fine motor skills, take blood pressure, auscultate heart sounds **Thorax:** Inspect thorax for shape and breathing effort, converse with patient about their breathing effort **Abdomen:** Take history on bowel function, inspect abdomen, auscultate abdomen for bowel sounds, abdominal palpation **Neurology:** Assess mental status, cranial nerves II, V and VII, tone and muscle strength in arms, sensation of touch under feet	Student initiates assessment through conversation. Performs suitable skills related to patient’s clinical and medical diagnosis. Takes head-to-toe approach, where patient’s symptoms are in focus for skills application. Student references prior clinical encounters to support clinical reasoning for performed assessment skills. The current patient encounter is a starting point for reflections, and reflections go beyond the actual assessment on the audio-recording.
Male 82 years old. Admitted to nursing home due to rehabilitation and mobilization and rehabilitation after cardiac arrest 14 days ago	Diabetes 2, hypertension, atrial fibrillation, atrial flutter, heart failure, anxiety, sleeping disorders, urinary retention, sacral pressure ulcer—fourth degree, heel ulcers on both feet, cardiac arrest—14 days ago, vertigo	**Heart and peripheral circulation:** Inspect extremities for skin colour/hair growth, palpate distal pulses, palpate for oedema, palpate and inspect capillary refill, assess fine motor skills, take blood pressure, auscultate heart sounds **Thorax:** Inspect thorax for shape, breathing effort, palpate thorax wall for thoracic expansion and vocal fremitus, percuss lungs, auscultate lungs, assess SpO_2_^2^ **Abdomen:** Inspect abdomen, auscultate abdomen for bowel sounds, abdominal palpation, percuss for kidney tenderness **Neurology:** Assess mental status, cranial nerves II, III, IV, VI, VII, VIII, IX, XI and XII	Student initiates assessment through conversation. Performs suitable skills related to patient’s clinical and medical diagnosis. Takes a head-to-toe approach where patient’s symptoms are in focus for skills application. Left out relevant assessments related to blood glucose The current patient encounter is a starting point for reflections, where the student mainly focuses on which B-PAS requires more practice. Gives rationale for performed assessments without further elaboration on why.
Female 87 years old. Admitted to nursing home for post-operative rehabilitation, mobilization and pain management after acute compression fracture in L4^4^ surgery	Hypertension, macular degeneration—10% eyesight, glaucoma, ischemic heart disease, osteoporosis, hiatus hernia, former ischemic cerebral insult and heart attack	**Heart and peripheral circulation:** Inspect extremities for skin colour/hair growth, palpate distal pulses, palpate for oedema, palpate and inspect capillary refill, assess fine motor skills, take blood pressure, auscultate heart sounds **Thorax:** Inspect thorax for shape and breathing effort, percuss the lungs, auscultate lungs, assess SpO2^2^ **Abdomen:** Take history on bowel function, inspect abdomen, auscultate abdomen for bowel sounds, abdominal palpation. **Neurology:** Assess mental status	Student initiates assessment through conversation. Performs suitable skills related to patient’s clinical and medical diagnosis. Takes a symptom-based approach through history-taking and conversation. The current patient encounter is a starting point for reflections, where the student mainly focuses on clinical reasoning for performed assessments.
Male 65 years old. Receives home nursing due to diabetic ulcer wound care on right foot	Diabetes 1, neuropathy	**Heart and peripheral circulation:** Inspect extremities for skin colour/hair growth, palpate distal pulses, palpate and assess distal pulses, palpate for oedema, evaluate extremities for skin sensation, assess fine motor skills on feet **Neurology:** Assess mental status	Student initiates assessment through conversation. Performs suitable skills related to patient’s clinical and medical diagnosis. Takes a symptom-based approach through history-taking and conversation. Student references prior clinical encounters to support clinical reasoning for performed assessment skills. The current patient encounter is a starting point for reflections, and reflections go beyond the actual assessment on the audio-recording.
Female 83 years old. Admitted to nursing home due to assessment of COPD^1^ exacerbation	COPD^**1**^	**Heart and peripheral circulation:** Inspect extremities for skin colour/hair growth, palpate distal pulses, palpate for oedema, palpate and inspect capillary refill, evaluate extremities for pain, take blood pressure, auscultate heart sounds **Thorax:** Take history on breathing effort, inspect thorax for shape and breathing effort, inspect thorax for skin colour/scar, auscultate lungs, assess SpO_2_^2^ **Abdomen:** Take history on bowel function **Neurology:** Assess mental status	Student initiates assessment through conversation. Performs suitable skills related to patient’s clinical and medical diagnosis. Takes a head-to-toe approach where patient’s symptoms are in focus for skills appliance. Student references prior clinical encounters to support clinical reasoning for performed assessment skills. The current patient encounter is a starting point for reflections, and reflections go beyond the actual assessment on the audio-recording.

^1^*COPD* Chronic obstructive pulmonary disease, ^*2*^*SpO*_*2*_ Blood oxygen level, ^*3*^*PCI* Percutaneous coronary intervention, ^*4*^*L4* Vertebrae number 4 of the lumbar spine

### Nursing students’ reflections during SRIs

Viewing the patient encounter and their own performance was the starting point for the students’ reflections during the SRI. The analysis resulted in several codes and two main categories: *perspectives on competent use of physical assessment in clinical rotation* and *key qualities of the learning environment* (Table 4).

**Table 4:** Factors influencing students’ use of physical assessment

Category	Code	Condensed meaning unit
Perspectives on competent use of physical assessments in clinical rotation	Perceived usefulness of performing physical assessment	Easier to cope with critical conditions
		Increased experiences that their assessments had impact
		Documentation made it easier to communicate findings
	Change of assessment approach	Transformation to a head-to-toe approach
		Transformation to a symptom-based approach
		Enhanced awareness of clinical reasoning processes
	Need for continuous practice of skills	Developing understanding of appropriate situations to use physical assessments
		Need to develop ability to recognize sounds
		Need to develop reasoning skills
		Expectation of own role
	Increased attention on communication as a part of physical assessment	Enhanced application of conversation as part of the assessment
		Enhanced effort to practice on own communication skills
	Ways of learning physical assessment skills in clinical rotation	Repetition of physical assessment skills appliance
		Active choice
		Stamina
		Defers physical assessment appliance
Key qualities of the learning environment	A safe and reflecting learning space	Engaged preceptors
		Safe to wear and use the stethoscope
		Missed opportunities
	Interprofessional collaboration	Collaboration with preceptors
		Expectation to perform physical assessments
		Collaboration with physicians
		Peer collaboration

### Category 1: perspectives on competent use of physical assessment in clinical rotation

The nursing students described their experiences using physical assessment during their final educational clinical rotation. Five codes were identified highlighting how they perceived their own use of physical assessment in clinical rotation: 1) perceived usefulness of performing physical assessment, 2) change of assessment approach, 3) need for continuous practice of skills, 4) increased attention on communication as part of physical assessment and 5) ways of learning physical assessment skills in clinical rotation.

#### Perceived usefulness of performing physical assessment

The students described various aspects related to using physical assessment in practice: these emphasized that a systematic approach was experienced as meaningful and valuable. They reported several, partly overlapping reasons. Practising physical assessment made it easier to cope with critical situations, as they could use a quick assessment approach (‘You can do some more assessments because the patient is feeling bad’, S9) and then move to a more thorough assessment, if necessary (‘We discovered something in our initial assessment, so we chose to perform a more thorough assessment’, S5). Using physical assessment also made it easier for students to communicate with other health care personnel, especially when communicating urgent messages. Here, one of the students referred to a situation where a physician, rather than waiting to see the patient the following day, changed his approach because of the student’s prompt and appropriate assessment (‘The physician came to see the patient quickly, and the patient was transported to the hospital right away’, S12). The students highlighted the value of being able to formulate, communicate and document findings from their physical assessment. Moreover, the systematic documentation gave the students a vocabulary with which to report data and subsequent actions in collaborative care (‘If you document, then it becomes more specific’, S2).

#### Change of assessment approach

The students were introduced to a physical assessment curriculum with skills systematized across organ systems in checklists. In the interviews, the students described using a combination of head-to-toe and a symptom-based approaches as a change of strategy from their prior clinical rotation. Several students emphasized that the head-to-toe approach ensured a complete in-depth assessment (‘Then there is less risk of missing something, S4). Consequently, the students were able to learn how to approach patients’ symptoms systematically (‘Because then I got to cram on how I can look at a patient’, S9), where they were also aided in articulating their observations (‘And what to call, to put names to things [assessments]’, S2). The students also described that the patient’s symptoms determined which assessment skills they performed (‘I’m going to use it more on a symptom-based approach and in relation to what the patient is admitted for’, S9). The symptom-based approach seemed to reflect students’ reasoning skills, when they reflected on why assessments were performed (‘I am much better now, at thinking over why I do things’, S1).

#### Need for continuous practice of skills

Over time, the students recognized that developing an understanding of how and when to perform physical assessment required continuous practice of skills (‘I am much better than before, but I’m still … Well yes, I need to practice more’, S4). Continuous practice was considered important, not only to develop the ability to listen to and recognize sounds, but also to perform the analytic activity of making distinctions and assumptions concerning the patient’s condition (‘I can maybe recognize crackles, stridor or wheezing, and where it is on the lungs’, S1). Another student described needing continuous practice in order to combine listening skills while trying to recognize possible pathological patterns and mechanisms behind symptoms—and to make tentative diagnostic hypotheses (‘Like with orthopnoea, I am beginning to know which assessments to perform and link it up to potential diagnoses, to know what the underlying conditions is’, S7). A more comprehensive understanding of the patient’s current health situation through physical assessment initiated a more targeted reasoning process (‘You are able to identify other types of data than reflected by the EWS’, S1). The students’ own ideas about what they would be expected to do following graduation became apparent (‘Everyone expects you to use it [the physical assessment skills]’, S9): one student, for example, described seeking different learning situations independently, trying to do as many of the tasks as possible that nurses are expected to do (‘Just to build confidence’, S9).

#### Increased attention on communication as a part of physical assessment

The students emphasized the importance of including conversation with the patient as a part of their assessments. When students took patients’ thoughts into consideration in their use of physical assessment, this demonstrated cue recognition for further in-depth assessment (‘Not only performing the assessments and then leaving but rather talking with the patient a bit more, assessing some more’, S7). Through eliciting patients’ expressed cues and concerns, the students learned to better ascertain whether the patient’s clinical conditions had deteriorated (‘Like the symptoms that a patient states, I am much better at recognizing that now’, S6). One student expressed increased awareness around how their own communication helped the patients express themselves more (‘Something more might come if I wait a bit longer and say “yes” or “mm”’, S2)—and how their sensitivity during the conversation could affect the patient response (‘Because once I start asking how she is feeling, it’s okay to give her time and space’, S2). This student further described a situation when it was easy to give patients input and then make their own conclusions on behalf of the patient. However, by practising leaving space between questions, the student gives patients more time to disclose their own matters of concerns or preference (‘Because it has to come from her. [...] I try not to put the words in her mouth’, S2).

#### Ways of learning physical assessment skills in clinical rotation

Regardless of how the students experienced the learning environment at different rotation cites, they all described strategies for using physical assessment skills on their own. By cultivating repetition and iterative skills application, the students developed a sense of habituation towards using physical assessment as an integrated part of their daily nursing (‘But if I do it and do it and do it, then it will gradually make more sense’, S2). One student described the inclusion of physical assessment as a natural part of nursing—a way of thinking (‘I believe it will be easier if you have this mindset of implementing physical assessment in all of your thinking’, S8). Stamina was highlighted in relation to skills practice (‘To push oneself, until you have applied it so many times that it becomes a habit’, S5). It was also described in relation to maintaining the motivation to practise specific skills—as stimulating continuous practice towards developing a repertoire of physical assessment skills (‘I have been conscious throughout this rotation, to auscultate and assess as many as possible’, S8). Persistent self-reflection around potential opportunities to perform physical assessment skills was also highlighted (‘Could I have done something here?’, S7).

In contrast to the above, some students expressed strategies to defer applying physical assessment skills during clinical rotation (‘It is a bit like, when I graduate, then I will start to take more responsibility’, S8), with an intention to perform physical assessment after graduation (‘Because then it will be easier’, S8). However, some of the students also noted that valuable practice opportunities were missed because they lacked understanding about the significance of physical assessment (‘If I had been able to do it from the start, I would have performed it [physical assessment] much earlier than I did’, S2).

### Category 2: key qualities of the learning environment

The students described the qualities of the learning environment as essential to their learning process. Two key qualities were 1: *a safe and reflective learning space,* and 2: *interprofessional collaboration*. All students highlighted these qualities as crucial for their skills acquisition and integration of physical assessment skills into their nursing routines.

#### A safe and reflective learning space

For the students, it was most important to feel part of a safe and reflective learning environment (‘To get support, reflect and talk about it [physical assessment skills]’, S8). In a safe learning environment, the stethoscope became a more natural and acceptable device to use (‘At least it hasn’t been strange to use the stethoscope here’, S2). The students reflected on how the stethoscope lowered the threshold for using physical assessment, enabling them to explore patient situations in-depth (‘I can start by examining, assessing the patient’, S5). For some students, it also seemed as if in-depth communication and the procedures that followed became easier or more natural when wearing and having the opportunity to use a stethoscope (‘And I notice that it is really positive that I can wear the stethoscope. I can ask additional questions as a natural part of my nursing routine’, S8). However, the students emphasized that the preceptors’ routines and ways of doing things influenced their own thinking and behaviour (‘We do get quite influenced by our preceptors’, S1); auscultation routines in particular seemed to be perceived as an indication of the students’ own use (‘I might have used it even more if there had been more nurses that uses it [the stethoscope]’, S6).

Some students did not have preceptors who integrated physical assessment skills in their daily nursing practice; these students found it difficult to perform more assessments than their preceptors typically did during patient encounters. One student referenced a patient encounter in which the preceptor was finishing up the visit (‘So it is a bit difficult in the sense of saying “I am just going to do this first”’, S7). In these instances, some students felt they needed to justify their suggestions or actions to preceptors who considered auscultation, palpation and percussion as being in the physicians’ domain rather than the nurses’ (‘I felt that they [the preceptors] looked at me in a peculiar way [ …] and they asked me when coming out to the car afterwards, “Why did you do that?”’, S5). These kinds of experiences seemed to be perceived as a prominent barrier to using and practising skills, and represent the opposite of a safe learning environment (‘I thought no, I won’t auscultate, because I feel uncomfortable’, S9).

#### Interprofessional collaboration

Collaboration with both nurses and physicians facilitated reflection around performed assessments in relation to patients’ treatment and care (‘There is more openness to have reflections on and discussions of assessments—what and why’, S5). These reflections and discussions seemed to build students’ confidence in their mastery of physical assessment skills (‘So, I have some knowledge, and I trust it’, S7). Students’ collaboration with preceptors also helped them understand how to integrate physical assessment in daily nursing (‘Because there is something about knowing in which clinical settings physical assessment are appropriate to perform’, S7). In particular, the students found it easier to apply physical assessment when they were met with an explicit expectation from preceptors or their educational institution (‘At this rotation site, you are asked specific questions such as, “Have you done this or that assessment?”’, S5).

In the nursing homes, some students experienced the physicians as gate-openers for using physical assessment (‘If it hadn’t been for them, I don’t think I would have been interested in becoming better at it’, S6). Here, the physicians invited them to be collaborators in the patients’ treatment and care (‘The physician asks if I can do assessments’, S6). Moreover, the physicians facilitated students’ reflection (‘What did we see, what did we feel, what did we hear, and what can that tell us?’, S9).

Learning with peers was also highly valued by the students. With their shared understanding of physical assessment from the educational programme, the students saw the value of practising assessment skills together (‘Then it was just the two of us’, S1). One student described how peers could assess the same patient, and then compare findings (‘Discuss what we heard [through auscultation]’, S8). This learning activity facilitated discussions about different techniques (‘Nina taught me a technique on how to palpate different pulses’, S8), and lastly helped them explore physical assessment skills application together (‘We motivated each other to use different assessment techniques’, S5).

## Discussion

This study reports on nursing student’s development and use of physical assessment during clinical rotation in their final educational year. Our main finding is that students’ rationales for performing specific physical assessment are based on the situation’s requirements. Additionally, we report on how students’ experiences of the learning environment influence their development and use of physical assessment skills. In the following, we emphasize how these findings relate to our previous study from a longitudinal perspective.

### Internalization of physical assessment in clinical nursing practice

A prominent finding in this study is that most of the students were able to determine which physical assessment skills were relevant in a given clinical encounter. Rather than using all the assessment skills they had learned, they took a flexible, integrated approach to the patients’ symptoms and concerns as a basis for performing assessments. The students’ reasons for why they used certain skills and not others largely reveal an internalization of physical assessment: here, their attention to the patients’ concerns or symptoms through cue recognition led to more targeted physical assessment appliance. This is in line with studies in both nursing and medical education, which have found that beginning learners tend to use more physical assessment skills than the situation requires [27, 28]; later in their education, students are able to cluster complex information and perform more targeted cue recognition [28].

The above finding contributes to a broader understanding of the literature, in which students’ decreased use of physical assessment skills is often related to an over-saturated physical assessment curriculum [12]. In our curriculum, we have given priority to assessment skills: circulatory, respiratory, abdominal and neurological systems, which may contribute to more effective use in nursing practice. One reason for why the students now demonstrated a more targeted use of assessment skills may be that they were in a process of learning higher-order thinking, with an increased ability to perform flexible and person-centred assessments. Furthermore, most students’ determination of which assessment skills to use was also more adequately adapted to the individual patient encounter and clinical context. This concurs with research showing that variations across specialities is found in both nurses’ and students’ practices [8, 9, 29].

Our finding that students experienced the conversation with patients to be a crucial part of their assessments reflects a person-centred approach. This aligns with Hafskjold et al. [30], who argue that dialogue is a feasible tool for eliciting and understanding patients’ cues and concerns. In this way, they can determine what currently is perceived as important by the patient and respond and act accordingly to the patient’s expressed needs—while also performing a holistic and systematic assessment.

### Nursing students’ learning process towards the internalization of physical assessment

Notably, the students highlighted a need for continuous skills practice. The insecurity we reported in Byermoen et al. [17] around performing specific physical assessment techniques was thus replaced with a desire to continuously refine their skills application. Further, the students highlighted the need to learn in which clinical settings they could integrate physical assessment in daily nursing practice. This exemplifies how the students’ experiential learning process evolved during their two final clinical rotations. This also concurs with research on how experiential learning supports higher levels of knowing when and how to integrate specific skills in practice [28].

Compared to our findings reported in Byermoen et al. [17] the students in this study used fewer physical assessment skills; however, their assessments were more targeted to the patients’ condition and the students acted on cue recognition. Three factors helped the students determine which assessments to use during the patient encounter: 1) increased integration of knowledge within human bioscience; 2) increased clinical reasoning skills; and 3) increased ability to transfer knowledge from prior experiences. This is seen in another study, as well, which report that students can develop a more attentive approach to cue recognition [31]. To know what is seen, heard and felt within the full context of the individual patient encounter, as well as knowledge developed through prior experience, are prerequisites for attentive cue recognition [28]. This emphasizes that, through experiential learning, students develop stronger reasoning skills when practising their acquired knowledge of cue recognition in clinical settings [32].

The students described an increased awareness of communication with patients as an integral part of their physical assessment strategy. This can be related to the students’ development of holistic attention towards the patient, where they are able to cluster information sources simultaneously, as described by Pearson [33]. Argyris and Schön [34] argue that this clustering of information works through mental mapping. Here, nursing students are guided in their cognitive reasoning processes through planning, executing and reflecting on their actions. These abilities represent a more advanced level of clinical competence. Furthermore, appropriately constituted mental maps can help students navigate the problem scenario, based upon prior knowledge and experience with similar challenges in the past; this can be seen as a part of nurses’ evidence-based practice [33]. Thus, combined with prior experiences from clinical encounters, the mental maps seemed to help students determine relevant assessments through a hypothetico-deductive strategy, such as hypothesis-thinking.

Malterud et al. [35] argue that the process of interpretative understanding has the potential to change clinical practice, when complementing hypothetico-deductive strategies by recognizing additional substantial modes of reflection. Studies report that educational programmes offering hypothetico-deductive strategies can contribute to an accelerated learning process [35–37]. Specifically, hypothetico-deductive learning activities may be structured into physical assessment explorations during simulation training. With hypothesis thinking, students’ previous experiences of assessment skills, knowledge in human-bioscience and nursing can be incorporated when complexity increases in their assessment findings. Simulation training with virtual patients can be a suitable learning activity where students integrate practical and theoretical knowledge through articulation and reflection [38].

### The value of a safe, stimulating and collaborative learning environment

The students described a safe and stimulating learning environment as a place where the health professionals working there expected them to perform physical assessment. Interestingly, students’ use of a stethoscope at the rotation site signalled that they perceived it as a safe environment in which to practise and use physical assessment skills. For example, a preceptor’s lack of auscultation routines, and on the other hand, a physician’s explicit request to perform an auscultation influenced whether the student’s felt comfortable performing the assessment. The stethoscope has traditionally been a tool used by physicians, rather than nurses [39], and this may explain the reason for students need to perceive expectations and thus acceptance from health professionals to use it in their practice [7].

The students also described that a safe learning environment involves interprofessional collaboration, in which they could use physical assessment skills whenever relevant. Moreover, a collaborative climate stimulated the students’ own motivation and persistence to continue practising physical assessment skills. In contrast, when the students did not perceive a safe learning environment, they would avoid performing specific physical assessment skills. This aligns with findings from other studies reporting that the lack of nursing students’ skills performance is related to the absence of role models and real opportunities to develop and practise skills [12, 40]. Indeed, support from the learning environment strongly influences how students develop the confidence to practise skills application and play a collaborative role in patient care, rather than a passive one [12].

Interprofessional as well as peer collaboration was emphasized by the students to facilitate reflective practices. Here, collaboration was experienced as having great value for their ongoing clinical reasoning skills and physical assessment skills development, when these were scaffolded through reflections. Reiser and Tabak [41] describe scaffolding as collaboration with a ‘more knowledgeable other’, which can help learners bridge their current knowledge with more sophisticated practice. In clinical encounters, collaboration with health professionals and peers provided opportunities for students to discuss skills, findings and possible interpretations; this, again, helped them develop higher-level abilities around clustering information and cue recognition. This finding concurs with prior research showing that collaboration initiates reflection through discussion with interprofessional teams or preceptors about students’ skills performance in clinical encounters [11, 32]. Interprofessional collaboration is also proposed as a significant component that provides students with opportunities to integrate theoretical and practice-based assessments [42].

Collaboration enables students to engage in different modes of reflection exercises that can help them achieve different goals through surface and deep learning, as Hattie and Donoghue [43] argue. In terms of developing physical assessment competence, surface learning is adaptive: for example, students learn both to perform physical assessment skills and interpret their findings. Achieving deep learning is more complex, and includes more conditional knowledge, such as the refinement of skills application, clinical reasoning and cue recognition in new contexts. Deep learning can be stimulated through students’ reflective practice around their own physical assessment performance via stimulated recall; this may represent a significant learning activity supporting students’ deep reflection on their own assumptions and/or performance. A reflective practice can further enable students to discover new ways of thinking and analysing clinical situations [44, 45].

The students in this study shared their ideas and experiences related to their own achievements, and how they could best learn while in clinical rotation. Most of them reported having engaged in a mode of reflection that Winne and Azevedo [46] describe as metacognition, which entails learning about learning. Thus, to increase nursing students’ reflexivity around their own learning, metacognition should be emphasized in their learning activities. This can help them become aware of and take responsibility for how they approach their own way of learning, while practising and developing their physical assessment skills. We also suggest implementing stimulated recall as a mandatory learning activity for students in physical assessment courses, scaffolded by faculty or preceptors, to provide deeper learning. Giving students the opportunity to discuss and reflect on their physical assessment performances while viewing their performance may prompt both surface and deep learning, as well as metacognition. By achieving deeper learning around their own physical assessment performance, nursing students can learn to extend these experiences to other contexts after graduation.

### Strengths and limitations

The nursing students participating in this study were motivated to refine and develop their physical assessment competence from the outset; as such, one may question whether they are representative of their nursing student cohort at the university. Moreover, the participants’ average age was slightly higher than the average at the university, which is 25 years. However, findings suggest that the students had similar experiences related to the use of physical assessments and learning environment during their clinical rotation, regardless of their overall competence or age.

The dual reflections occurring as part of the SRI may be biased in instances where the researcher addressed other topics than those the students intended to address. However, we can assume this approach helped the students identify aspects that deserved further elaboration through reflective practice.

Rigor, an important consideration in a qualitative study [47], was ensured by the thorough iterative phenomenological hermeneutical approach, and by a complementary research team. The researchers who participated in the data analysis and interpretation have different backgrounds within nursing science, communication, health services research, ethics and educational studies, and included both women and men. This strengthened the reflexibility of the study, reduced researcher bias and ensured that the students’ perspectives and experiences were understood in-depth.

Another strength of the study is that its findings on students’ use of physical assessment skills are transferrable to international settings within physical assessment courses in nursing education. Moreover, this paper describes different processes influencing nursing students’ experiences around their development of physical assessment competence: this, too, is relevant across skills education and levels.

## Conclusion and implication for practice

This study offers a novel, comprehensive and in-depth description of what influenced nursing students’ learning processes of using physical assessment skills during clinical rotation. Our study demonstrates the importance of designing and implementing appropriate learning activities that facilitate nursing students’ development of physical assessment skills. The results reveal that integrating physical assessment skills training in daily nursing practice involves a complex personal learning transition—starting from a checklist-oriented approach considering all learned skills, to using communication and a symptom-based approach to perform targeted assessments. Therefore, there is need for introducing and scaffolding physical assessment skills as an integral part of theoretical and clinical rotation curriculum development. We suggest that nursing education implement compulsory learning activities that explicitly aims at supporting nursing students’ ability to articulate their theoretical understanding – through reflective practices and clinical reasoning processes with peers, preceptors and faculty.

A safe learning environment at the clinical rotation site enabling collaborative learning, interprofessional communication and reflective practices is key in fostering development of nursing students’ physical assessment skills. Educational research can further explore nursing students’ learning processes of using physical assessments in different clinical contexts, such as in the setting of specialist health services, where students collaborate more closely with several professions in their learning processes, offering locally available and qualified support in the use of physical assessment skills.

Systematically nurturing nursing students’ physical assessment competence and clinical reasoning early in their education may lead to more confident newly graduated nurses—who, in turn, will contribute much-needed competence that enhance patient safety at all levels of health care.

In this study, we have employed stimulated recall as a research method to directly observe and record how students actually perform physical assessment skills. The findings elicit how stimulated recall makes nursing students reflect in ways that incorporate the complexity of their patient observations, previous experiences of nursing, learned assessment skills, knowledge in human-bioscience and their immediate assessments, into an integrated hypothesis creation and learning process. We propose stimulated recall as a novel reflective learning activity to foster students’ clinical reasoning and metacognition skills and achieve deep learning.

## Data Availability

Datasets used during the current study are available from the corresponding author on request.

## Abbreviations

EWS: Early warning score
NSD: The norwegian centre for research data
SRI: Stimulated recall interview
PAS: Physical assessment skills
SpO_2_: Blood oxygen level
CNI-XII: Cranial nerves numbers 1–12
COPD: Chronic obstructive pulmonary disease
PCI: Percutaneous coronary intervention
L4: Vertebrae number 4 of the lumbar spine
